# Headache in patients with non-functioning pituitary adenoma before and after transsphenoidal surgery – a prospective study

**DOI:** 10.1007/s11102-024-01401-3

**Published:** 2024-05-20

**Authors:** Victor Hantelius, Oskar Ragnarsson, Gudmundur Johannsson, Daniel S. Olsson, Sofie Jakobsson, Erik Thurin, Dan Farahmand, Thomas Skoglund, Tobias Hallen

**Affiliations:** 1 Department of Internal Medicine and Clinical Nutrition, Institute of Medicine, Sahlgrenska Academy, University of Gothenburg, Gothenburg, Sweden; 2 Department of Medicine, Sahlgrenska University Hospital, Gothenburg, Sweden; 3 Wallenberg Center for Molecular and Translational Medicine, University of Gothenburg, Gothenburg, Sweden; 4 Cardiovascular, Renal and Metabolism (CVRM), Late-Stage Clinical Development, BioPharmaceuticals R&D, AstraZeneca, Gothenburg, Sweden; 5 Institute of Health and Care Sciences, The Sahlgrenska Academy, University of Gothenburg, Gothenburg, Sweden; 6 Department of Clinical Neuroscience, Institute of Neuroscience and Physiology, Sahlgrenska Academy, University of Gothenburg, Gothenburg, Sweden; 7 Department of Radiology, Sahlgrenska University Hospital, Gothenburg, Sweden; 8 Department of Neurosurgery, Sahlgrenska University Hospital, Gothenburg, Sweden

**Keywords:** Quality of life, Non-functioning pituitary adenoma, Transsphenoidal, Surgery, Headache, Pituitary tumor

## Abstract

**Purpose:**

To study the long-term effect of transsphenoidal surgery (TSS) on headache in patients with non-functioning pituitary adenoma (NFPA) and identify factors predicting headache relief following TSS.

**Methods:**

We evaluated headache in 101 consecutive patients with NFPA who underwent TSS from September 2015 to December 2021, preoperatively and 12-months post-surgery, by using the Migraine Disability Assessment (MIDAS) questionnaire. Health-related quality of life (QoL) was assessed using the EQ-5D visual analogue scale (EQ-VAS).

**Results:**

Of 101 patients, 27 (27%) experienced disabling preoperative headache. Among these, the median total MIDAS score improved from 60 (interquartile range (IQR): 19–140) to 10 (IQR: 0–49) (*P* = 0.004). Additionally, headache frequency over a 90-day period decreased from 45 (IQR: 25–83) to 6 (IQR: 3–36) days (*P* = 0.002), and headache intensity decreased from 5 (IQR: 4–7) to 4 (IQR: 2–7) (*P* = 0.016) at 12-months post-surgery. At 12 months post-surgery, 18 (67%) of 27 patients with preoperatively disabling headache showed clinically relevant improvement of their headache, 4 (15%) showed deterioration, and 5 (19%) remained unchanged. In patients with clinically relevant improvement of their headache, the EQ-VAS score improved from 50 (IQR: 30 − 7) to 80 (IQR: 65–86) (*P* < 0.001). Of the 74 patients with no preoperative headache, 11 (15%) developed postoperative headache. We identified no clinical factors predicting postoperative headache relief.

**Conclusion:**

The study supports that clinically significant and long-lasting improvements of disabling headache and QoL can be achieved with TSS in a substantial number of patients with NFPA.

## Introduction

Pituitary tumors comprise approximately 15% of all intracranial tumors [1]. Although most are benign non-functioning pituitary adenomas (NFPAs), many require surgical treatment [2], with transsphenoidal surgery (TSS) as primary treatment [3].

In addition to visual-field defects from compression of the optic chiasm, headache is the most common symptom in NFPA [4], with a prevalence ranging from 33 to 72% [5]. Headache is also the most common symptom leading to investigation with computed tomography (CT) or magnetic resonance imaging (MRI) of the head, and discovery of a pituitary tumor [6]. Although pituitary tumor-associated headache is related to impaired quality of life (QoL) [7, 8], headache is also a common symptom with many different etiologies across the general adult population [9]. Thus, it is difficult to distinguish headache associated with pituitary tumors from common headache in the general population, and consequently confirm the effect of TSS on headache symptoms. Although previous studies support TSS for patients with pituitary adenomas and intractable headache [10, 11], headache alone is generally not considered a sufficient indication for surgery [12].

Although several studies report significant improvement of headache following TSS, most of them are limited by small cohorts, often representing various adenoma etiologies or a short post-surgical follow-up time [7, 8, 13]. A previous study on 110 patients with both NFPA and functioning pituitary adenomas, based on the Gothenburg Pituitary Tumor Study (GoPT) [14], reported improvements in disabling headache at 6-months following TSS [15]. Another recent prospective study on 122 patients with both NFPA and functioning pituitary adenomas, and the same follow-up time, reported comparable results [16].

In this study, our aim was to evaluate the long-term effect of TSS on headache in a cohort including only patients with NFPA. Furthermore, we aimed to identify factors capable of predicting headache relief following TSS.

## Materials and methods

### Study design

Data were obtained from the GoPT study, which was a prospective study of consecutive patients undergoing endoscopic TSS for a pituitary tumor at Sahlgrenska University Hospital from September 2015 to December 2021. Patient health status was assessed preoperatively and at 12-months following TSS [14].

### Patients

Of 228 patients considered for study inclusion, 154 were diagnosed with NFPA. Of these, 27 declined to participate, 20 did not complete their 12-month visit, two discontinued their participation in the study, and four died within 12 months following TSS. The remaining 101 (66%) patients included in the analysis completed the Migraine Disability Assessment (MIDAS) and EQ-5D questionnaires both before and at 12-months post-surgery.

### Ethics

This study complied with the guidelines set forth by the World Medical Association Declaration of Helsinki. Each patient provided written informed consent prior to participation in the study. The study was approved by the Regional Research Ethics Committee in Gothenburg, Sweden (Reference number: 387 − 15).

### MIDAS and criteria for headache classification

The MIDAS questionnaire was used to evaluate headache in patients before and at 12-months post-surgery. MIDAS is a self-administered questionnaire and considered to provide a valid estimation of headache [17]. Because reporting is equivalent to diary measures [18] and correlates with physician judgment of patient pain and disability [19], MIDAS is considered a tool capable of measuring treatment-specific headache efficacy [18, 20].

The first five questions measure the impact of headache on daily living by evaluating the number of days during a 3-month period that headache resulted in the following: (1) missed work, (2) 50% reduction in work productivity (apart from the days from question 1), (3) inability to perform household tasks, (4) 50% reduction in household productivity (apart from the days from question 3), and (5) inability to participate in family and social activities. Patient responses result in a score (maximum score: 270) that grades patient disability as follows for each question: Grade I (0–5), “little to no disability”; Grade II (6–10) “mild disability”; Grade III (11–20), “moderate disability”; and Grade IV (21 or higher) “severe disability”. A MIDAS grade ≥ II suggests a headache as disabling [18]. The questionnaire also measures headache frequency from 0 90 and intensity on a 11-point scale, with 10 representing severe pain. We defined a 50% reduction in MIDAS score as a clinically relevant improvement based on previous studies [12, 13, 21]. Similarly, a ≥ 50% increase in score was considered a clinically relevant deterioration in symptoms.

Patients who experienced a clinically relevant reduction in headache after TSS were considered to have had pituitary tumor-associated headache according to criteria established by the International Classification of Headache Disorders (ICHD)-3 [22].

### QoL assessment

Health-related QoL was assessed using the Swedish version of the EQ-5D and its visual analogue scale (EQ-VAS) [23]. Each patient graded their perceived health status before and at 12-months after TSS on a 101-point scale (0 = “the worst health you can imagine”; 100 = “the best health you can imagine”). The EQ-5D is commonly used by clinical researchers to evaluate QoL [24] and treatment effects, including those in patients with pituitary tumors [25, 26] and headache [27, 28].

### Patient and tumor characteristics

All patients underwent preoperative assessment by an endocrinologist that included evaluation of anterior pituitary function through clinical and biochemical evaluation. Patients with functioning pituitary adenomas were excluded.

All patients underwent MRI (Achieva 3.0T using gadolinium-contrast-enhanced T1- and T2-weighted sequences; Philips Healthcare, Andover, MA, USA), except for two patients who underwent a computed tomography scans prior to surgery. All images were evaluated by the same radiologist. The variables of interest were size (micro- versus macroadenoma, 1 cm), cavernous sinus invasion (Knosp $\ge$3), and compression of the optic chiasm.

After surgery, tumor tissues from all patients underwent routine histopathologic analysis at the Sahlgrenska University Hospital for classification according to World Health Organization guidelines [29]. Perioperative and postoperative leakage of cerebrospinal fluid (CSF) was recorded, with postoperative leakage defined as leakage requiring lumbar drainage and/or reoperation. All patients underwent postoperative MRI approximately 6 months following surgery for evaluation of a residual tumor.

### Statistical analysis

Categorical data are presented as numbers and percentages, normally distributed data are presented as mean ± standard deviation (SD), and non-normally distributed data as the median and interquartile range (IQR). Fisher’s exact test was used to determine associations between categorical data. Continuous data were compared between groups using the Mann–Whitney *U* test for non-normally distributed data and Student’s *t* test for normally distributed data. The Wilcoxon signed-rank test was used for within-group comparisons (headache data and EQ-VAS results before and at 12-months post-surgery).

Uni- and multivariable logistic regression was used to identify possible factors predictive of disabling headaches prior to surgery [i.e., sex, age, body mass index (BMI), Ki-67 level, cavernous sinus invasion, and compression of the optic chiasm]. Logistic regression analysis was used to assess the influence of these factors on the effect of TSS in patients with disabling headache (improved vs. not improved). Analyses were performed using SPSS (v.29.0; IBM Corp., Armonk, NY, USA).

## Results

### Baseline measurements

Of 101 patients, 60 (59%) were men and 41 (41%) were women. The mean age was 62 ± 14 years, and the mean BMI was 27 ± 5 kg/m^2^. One patient had an NFPA with the largest dimension less than 1 cm. Invasion of the cavernous sinus was observed in 39 (39%) patients and compression of the optic chiasm in 91 (90%) patients (Table 1).

**Table 1:** Characteristics of the study cohort (*n* = 101)

Male	60 (59)
Female	41 (41)
Age, years	62 ± 14
BMI, kg/m^2^	27 ± 5
Macroadenoma (tumor size > 1 cm)	100 (99)
Cavernous sinus invasion	39 (39)
Compression of the optic chiasm	91 (90)
Ki-67 > 3%	7 (7)

Data are presented as the mean ± standard deviation, or *n* (%)

Ki-67 is a protein used as a marker for cell proliferation where an index over 3% has been associated with more rapid tumor growth, missing in two cases

*Abbreviations* BMI, body mass index

### Headache before surgery

Of the 101 patients, 60 (59%) experienced some form of headache 3-months prior to surgery, with seven (7%) reporting a daily headache during that period. Twenty-seven (27%) patients had disabling headache according to MIDAS grading, with 20 having severe disabling headache (Grade IV), one moderate disabling headache (Grade III), and six mild disabling headache (Grade II). The remaining 74 (73%) reported little or no headache. The median MIDAS score among all included patients was 0 (IQR: 0–10 days), with median headache frequency over a 90-day period at four days (IQR: 0–20 days) and median intensity at 2 (IQR: 0–5). Among the 27 patients with disabling headache, the median MIDAS score was 60 (IQR: 19–140), headache frequency over a 90-day period at 45 days (IQR: 25–83 days) and intensity at 5 (IQR: 4–7).

### Predictors of headache before surgery

Univariable analysis of possible predictors of headache before surgery showed that patients with disabling headache were younger (*P* < 0.001), were more likely to harbor a tumor with Ki-67 > 3% (*P* = 0.001) but less likely to have compression of the optic chiasm (*P* = 0.021) (Table 2).

Multivariable logistic regression analysis including the three significantly associated variables (age, compression of the optic chiasm and Ki-67) from the univariable analysis revealed only younger age to be associated with disabling headache (*P* = 0.003).

**Table 2:** Patient characteristics before surgery according to MIDAS grade (*n* = 101)

	No disabling headache (Grade I) (*n* = 74)	Disabling headache (Grade II–IV) (*n* = 27)	*P*
Sex			0.37
Men	46 (62)	14 (52)	
Women	28 (38)	13 (48)	
Age, years	65 ± 12	53 ± 15	< 0.001
BMI	27 ± 4	29 ± 7	0.24
Cavernous sinus invasion	28 (38)	11 (41)	0.82
Compression of chiasm	70 (95)	21(78)	0.021
Ki-67 > 3%	1 (1)	6 (23)	0.001
Any preoperative deficiency	43 (58)	17 (63)	0.82
Adrenal insufficiency	18 (24)	8 (30)	0.61
Central hypothyroidism	28 (38)	9 (33)	0.82
Growth hormone deficiency	15 (20)	8 (30)	0.42
Diabetes insipidus	2 (3)	2 (7)	0.29
HH	34 (45)	11 (41)	0.66

Data are presented as the mean ± standard deviation, or *n* (%)

Ki-67, missing in two cases

*Abbreviations* BMI, body mass index; HH, Hypogonadotropic hypogonadism

### Headache after surgery

**Fig. 1:**
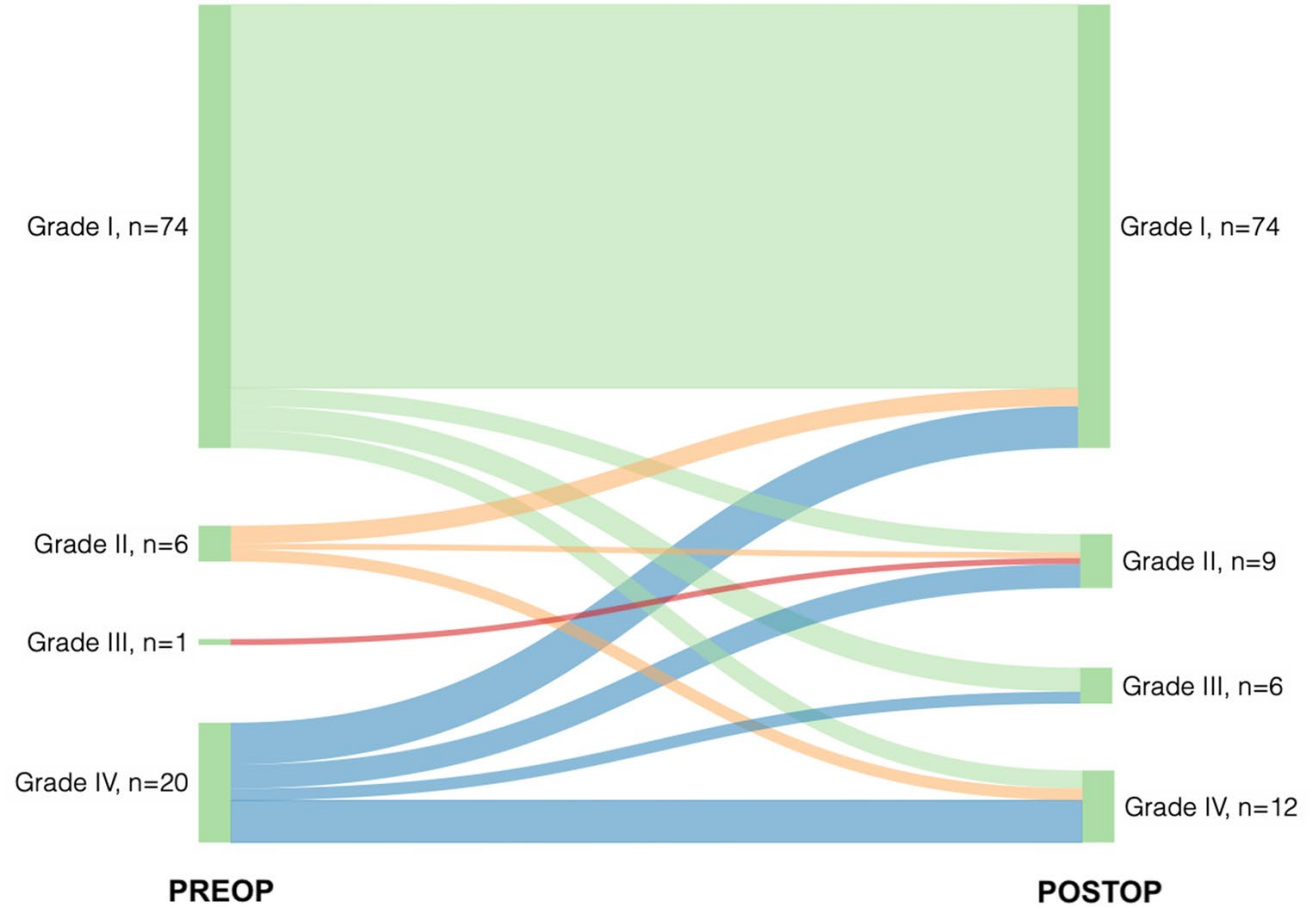
Sankey diagram created using SankeyMATIC showing MIDAS grade categories before (PREOP) and at 12-months post-surgery (POSTOP). Grade I, “little to no disability”; Grade II, “mild disability”; Grade III, “moderate disability”; and Grade IV, “severe disability”. *Abbreviations* POSTOP, postoperation; PREOP, preoperation

After surgery, 27 (27%) reported disabling headache according to MIDAS grading, with 12 (44%) patients reporting severe disabling headache (Grade IV), six (22%) moderate disabling headache (Grade III), and nine (33%) mild disabling headache (Grade II). The remaining 74 (73%) patients reported little or no headache (Fig. 1).

**Fig. 2:**
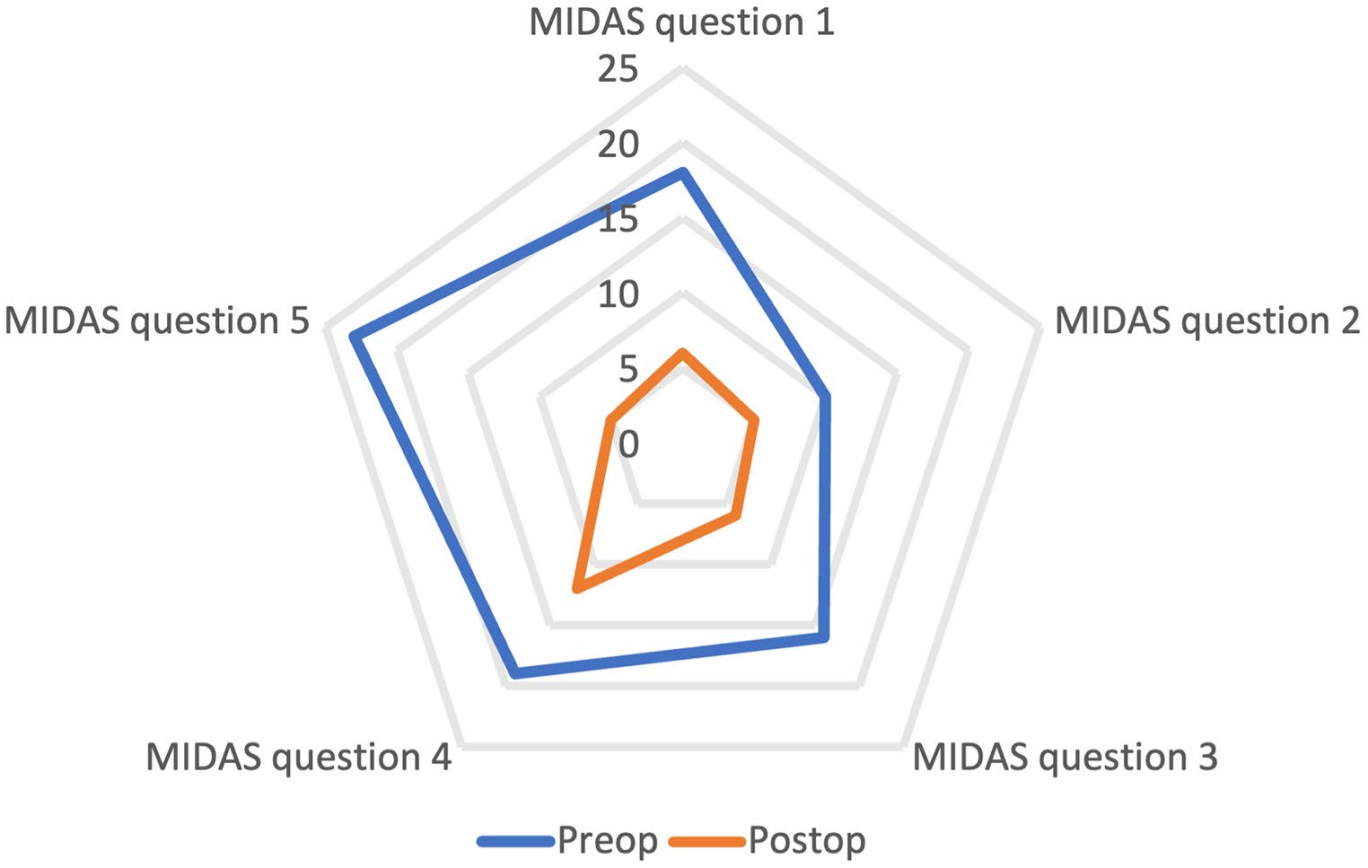
Mean pre- and postoperative total MIDAS scores according to the five questions used for MIDAS grading of the headaches´ impact on daily life in patients reporting preoperative disabling headache (*n* = 27). Responses conform to the number of days with headache over a 90-day period resulting in: (1) missed work, (2) 50% reduction in work productivity, (3) inability to perform household tasks, (4) 50% reduction in household productivity, and (5) inability to participate in family and social activities. *Abbreviations* MIDAS, Migraine Disability Assessment; Postop, postoperation; Preop, preoperation

**Fig. 3:**
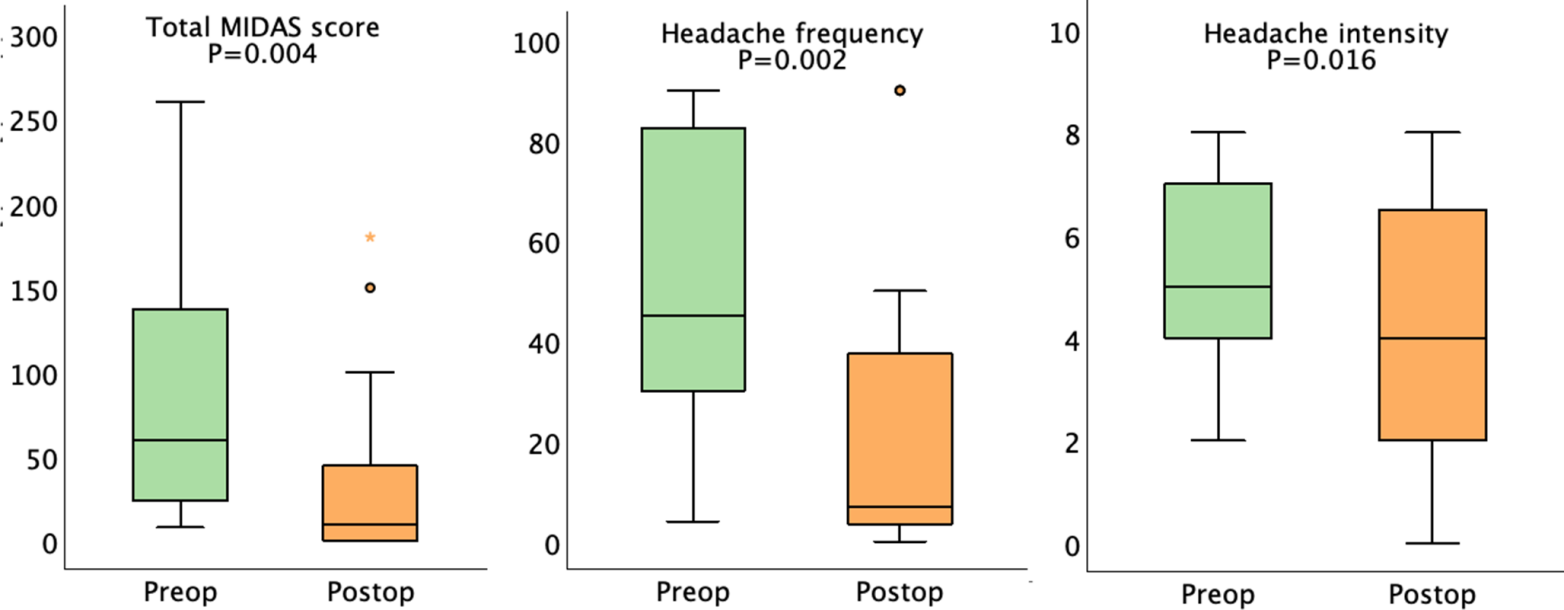
Median (IQR) total MIDAS score, headache intensity, and headache frequency before surgery and at 12-months post-surgery in the 27 patients with preoperative disabling headache. *Abbreviations* IQR, interquartile range; MIDAS, Migraine Disability Assessment; Postop, postoperative; Preop, preoperative

For the entire cohort, the median (IQR) headache frequency decreased from 4 (0–20) days before surgery to 2 (0–9) days (*P* = 0.028) at 12-months post-surgery, with no changes in MIDAS score or headache intensity. However, among the 27 patients reporting disabling headache before surgery, the total MIDAS score decreased from 60 (19–140) before surgery to 10 (0–49) at 12-months post-surgery (*P* = 0.004). The mean score for the five MIDAS questions (Q1-Q5) were before surgery: Q1 = 18, Q2 = 10, Q3 = 16, Q4 = 19 and Q5 = 23. At 12-months after surgery all mean scores had improved: Q1 = 6 (*P* = 0.008), Q2 = 5 (*P* = 0.193), Q3 = 6 (*P* = 0.013), Q4 = 12 (*P* = 0.178) and Q5 = 5 (*P* = 0.008) (Fig. 2). Furthermore, headache frequency and intensity decreased from 45 (25–83) days to 6 (3–36) days (*P* = 0.002) and from 5 (4–7) days to 4 (2–7) days (*P* = 0.016), respectively (Fig. 3).

**Fig. 4:**
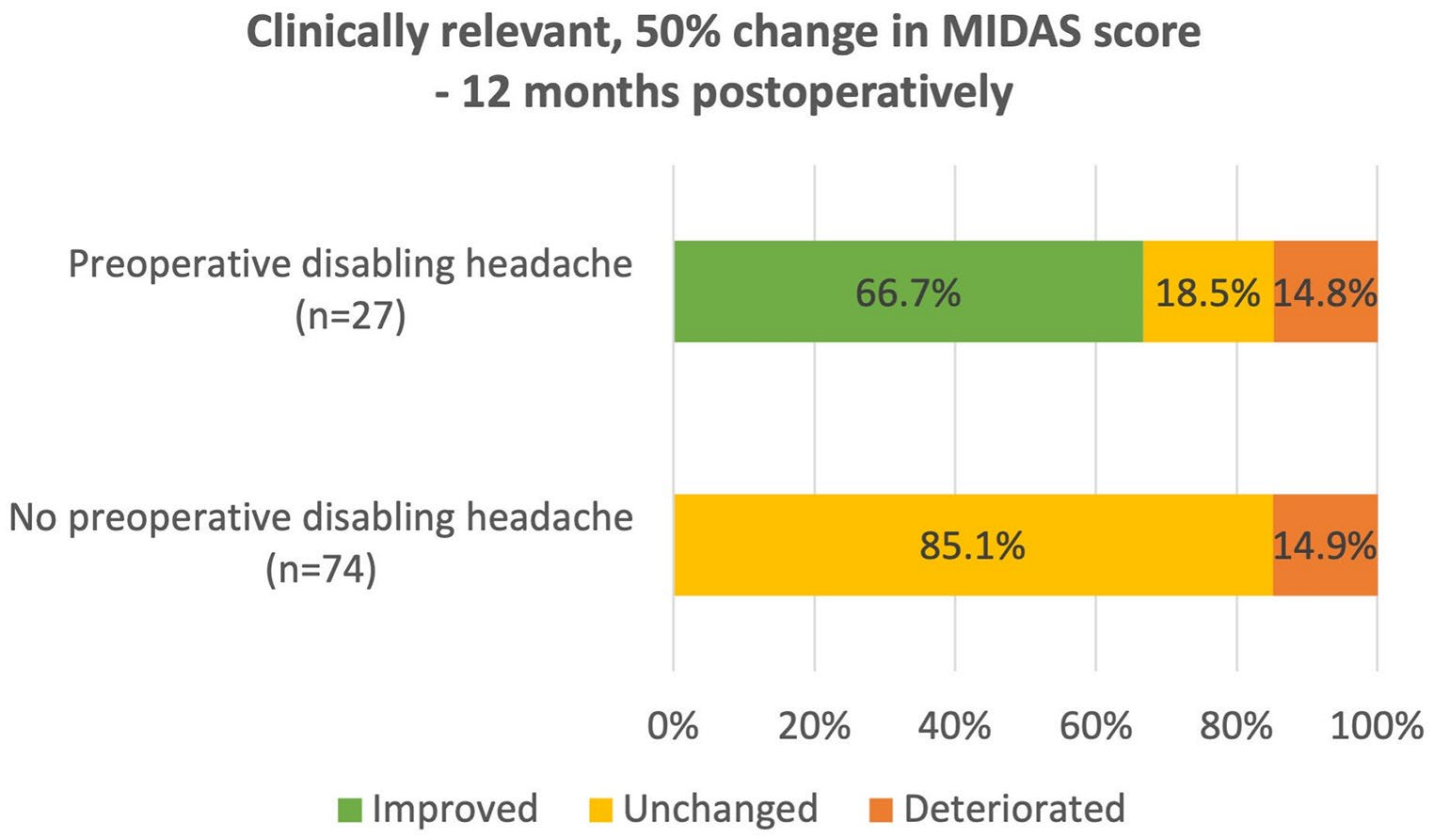
Clinically relevant changes in total MIDAS score before and at 12-months post-surgery in patients with no preoperative disabling headache (*n* = 74) and those with preoperative disabling headache (*n* = 27). *Abbreviations* MIDAS, Migraine Disability Assessment

Among the 27 patients with disabling headache prior to surgery, 18 (66.7%) had clinically relevant improvement at 12 months following TSS, four (14.8%) had clinically relevant deterioration, and five (18.5%) were unchanged (Fig. 4). Among the 74 patients with no disabling headache prior to surgery, 11 (14.9%) showed clinically relevant deterioration following surgery leading to disabling headache (Fig. 4).

### Factors predicting clinically relevant reduction in headache

Univariable logistic regression analysis demonstrated no associations between clinically relevant improvement of headache with neither sex, age, cavernous sinus invasion, BMI, compression of the optic chiasm, CSF leakage, postoperative residual tumor or any hormone deficiency including diabetes insipidus after surgery (Table 3).

**Table 3:** Patient characteristics according to relevant improvement in headache (*n* = 27)

	Improved (*n* = 18)	No improvement (*n* = 9)	OR (95% CI)	*P*
Sex				
Men	9 (50)	5 (56)	*0.80 (0.16–3.99)*	*0.79*
Women	9 (50)	4 (44)		
Age, years	52 ± 14	53 ± 13	1.00 (0.94–1.06)	0.87
BMI	30 ± 7	27 ± 6	1.07 (0.92–1.23)	0.39
Compression of optic chiasm	14 (78)	7 (78)	1.00 (0.15–6.85)	1.00
Cavernous sinus invasion	7 (38)	4 (44)	0.80 (0.16–4.02	0.78
CSF leak, perioperative	8 (44)	3 (33)	0.80 (0.16–4.02)	0.78
CSF leak, postoperative	1 (6)	0 (0)	-	1.00
Residual tumor > 5 mm	4 (22)	5 (56)	0.31 (0.06–1.64)	0.18
Any postoperative deficiency	12 (67)	8 (89)	0.25 (0.03–2.49)	0.24
- Adrenal insufficiency	6 (33)	3 (33)	1.00 (0.18–5.46)	1.00
- Central hypothyroidism	7 (39)	7 (78)	0.18 (0.03–1.14)	0.07
- GH deficiency	11 (61)	7 (78)	0.45 (0.07–2.81)	0.39
- Diabetes insipidus	2 (11)	3 (33)	0.25 (0.03–1.89)	0.18
- HH	6 (33)	5 (55)	0.40 (0.08–2.06)	0.27

Data are presented as the mean ± SD or *n* (%)

*Abbreviations* BMI, body mass index; CI, confidence interval; CSF, cerebrospinal fluid; GH, growth hormone; HH, Hypogonadotropic hypogonadism; OR, odds ratio; SD, standard deviation

### Pre- and postoperative QoL and the association with headache

**Fig. 5:**
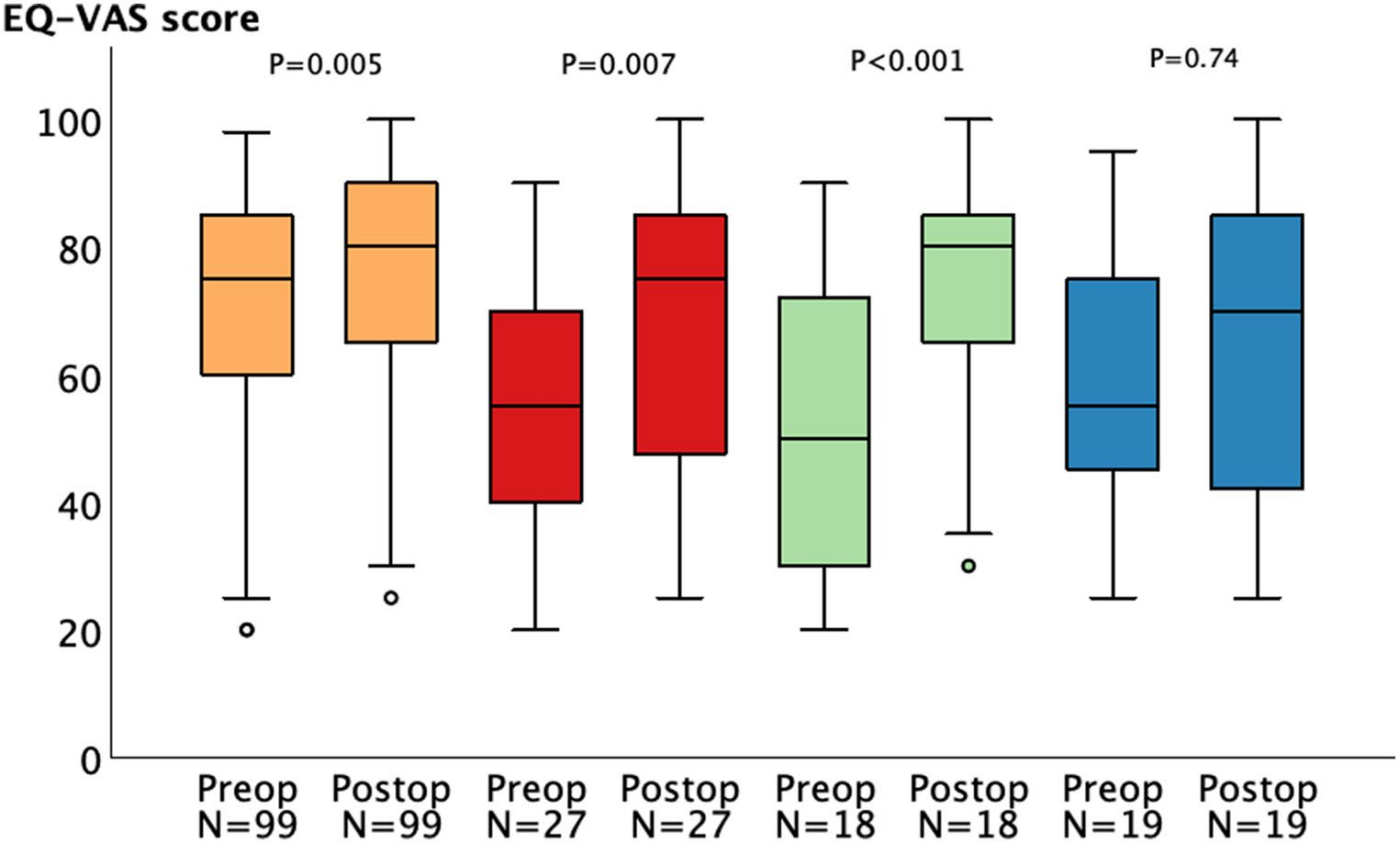
Median (IQR) EQ-VAS scores before and at 12-months post-surgery. Color coding: orange, the entire cohort with 2 missing cases (*n* = 99); red, patients reporting preoperative disabling headache (*n* = 27); green, patients reporting clinically relevant improvement in their headache post-surgery (*n* = 18); and blue, patients reporting deterioration, no improvement, or development of a new headache with 1 missing case (*n* = 19). *Abbreviations* EQ-VAS, EQ-5D visual analogue scale; IQR, interquartile range; Postop, postoperative; Preop, preoperative

For the entire cohort, median (IQR) EQ-VAS scores increased from 75 (60–85) before surgery to 80 (65–90) at 12-months post-surgery (*P* = 0.005). In patients reporting disabling headache before surgery (*n* = 27), the median EQ-VAS score increased from 55 (40–70) to 75 (40–85) (*P* = 0.007) The Eq. 5D-VAS scores improved from 50 (30–74) to 80 (65–86) (*P* < 0.001) in patients reporting clinically relevant improvement in their headache (*n* = 18). In those patients reporting deteriorated headache symptoms, had no relevant improvement in headache, or developed new headache symptoms (*n* = 20), there was no significant change in the median EQ-VAS score post-surgery [55 (40–75) before surgery and 70 (39–85) at 12-months post-surgery; *P* = 0.74] (Fig. 5).

## Discussion

In this prospective study we have evaluated headache by using the MIDAS questionnaire before and at 12-months after TSS in 101 patients with NFPA. We found that 67% of patients reporting disabling headache prior to surgery had a clinically relevant improvement whereas 15% of patients without preoperative disabling headache developed headache post-surgery. Among patients who reported disabling headache before surgery, there were improvements in headache frequency and intensity, as well as in the MIDAS score, at 12 months post-surgery. In these patients, as well as for the cohort as a whole, the QoL improved 12 months after surgery. However, patients who reported deterioration, no improvement, or the development of new headaches did not experience any improvement in QoL.

Although these results agree with two previous prospective studies of similar sizes [15, 16], the present study focused on a more homogenous cohort comprising only patients with NFPAs and across a longer follow-up time of 12 months. Other smaller studies have also reported similar findings, two of them using headache evaluations before and 6-months after surgery using the Headache Impact Test (HIT)-6 [7, 8]. One previous study had an 18-month follow-up period including patients with both functioning and NFPAs, which was noted as a study limitation [13].

Our results showed that younger age and Ki-67 > 3% was associated with disabling headache before surgery in univariable analysis. Other previous studies of patients with pituitary tumors and headache have shown similar relationships between patient age [2, 12, 15, 30], age and Ki-67 level [13, 31]; however, in our multivariable analysis, only younger age was independently associated with headache.

The association between headache and younger age could be partially explained by the MIDAS questionnaire’s emphasis on the impact of headaches on working ability, as younger patients are more likely to be below retirement age. Moreover, the association between Ki-67 as a proliferative marker with headache may be explained by the relationship of different headache-specific pathophysiological mechanisms with more progressive pituitary tumors. Furthermore, other studies have identified increased intracranial pressure due to mass effect, as well as pain-receptor activation due to intrasellar pressure, as possible causes of pituitary tumor-associated headache [10, 32]. Other studies suggest a more multifactorial mechanism, including cavernous sinus invasion, dural stretch, compression of the optic chiasm, cystic or solid mass of tumors, and hormone hypersecretion [5, 33].

It has been suggested that tumor extension rather than tumor size might cause headache [5, 10, 34]. An intact sellar diaphragm and cavernous sinus has the potential of increasing the intrasellar pressure [5, 10, 35], although headache might also develop secondary to more extensive and infiltrative growth [5]. In this study we chose to define tumor configuration as more extensive upward tumor growth if compression of the optic chiasm was present and lateral extension as cavernous sinus invasion. We observed that patients diagnosed with optic chiasm compression reported disabling headaches less frequently. This phenomenon could be explained by patients seeking care earlier due to headaches, leading to the diagnosis of NFPA before symptoms from optic chiasm compression occur. Another possible explanation may be a reduction in intrasellar pressure resulting from when tumor growth causes penetration of the sellar diaphragm. No association between grade of cavernous sinus invasion and preoperative headache was, however, found.

Considering the high prevalence of headaches in the general population [9], it is improbable that every case of reported disabling headache is associated with a pituitary tumor. However, our findings of improved headache post-surgery in a significant number of patients suggest that their status according to the International Classification of Headache Disorders ICHD-3 criteria [22] qualified them as presenting pituitary tumor-associated headache prior to surgery. Our results agree with previous studies showing similar post-surgical improvements (49–81%) [10, 15, 30, 36] and deteriorations in symptoms (8–17%) [7, 15, 36]. These findings offer important information that should be provided to patients during preoperative assessment regarding possible improvement, deterioration, or development of new headache after TSS.

Discovery of factors associated with improvement of headache after TSS would be beneficial for preoperative clinical decision-making. However, in this study no such predictive factors were found in regression analyses, including preserved hormonal function or presence of postoperative residual tumor.

Previous studies suggest that headache, both before and after TSS adversely affects patients through reduced QoL [8, 18]. In the present study, patients reporting a clinically relevant improvement in their headache also showed significant improvement in their QoL at 12-months post-surgery. The patients that reported deteriorated symptoms, no improvement, or development of a new headache post-surgery, did not show significant improvement in their QoL.

This study has limitations. These include the relatively small number of patients reporting disabling headache, which made obtaining statistical significance and analytical power difficult and complicated the identification of predictive factors. Additionally, because patients with microadenomas rarely meet the criteria for TSS, we did not assess tumor size according to volume rather than classifying them as either a micro- or macroadenoma. Furthermore, although MIDAS and HIT-6 are both accepted as clinically accurate instruments for measuring headache, it is possible that headache intensity can more significantly influence HIT-6 scores, whereas headache frequency may disproportionately influence MIDAS scores [37].

Strengths of the study include its prospective design and a homogenous cohort of patients with the same diagnosis, frequently lacking in previous studies [7]. By focusing exclusively on NFPA patients we increase the likelihood that observed postoperative changes in headache are related to the tumor itself, rather than a being consequences changes in of hormone overproduction. Additionally, this study offers information across a longer time period (12-months after TSS) compared to most previous studies.

## Conclusion

In conclusion, TSS may be beneficial and result in long-lasting improvement of tumor-associated disabling headache in subset of patients with NFPA. Further studies are required to identify factors capable of predicting which patients are likely to benefit from surgery.

## Data Availability

Some or all datasets generated during and/or analyzed during the current study are not publicly available but are available from the corresponding author on reasonable request.

## References

[1] Melmed S (2020) Pituitary-Tumor Endocrinopathies. N Engl J Med 382:937–950 32130815 10.1056/NEJMra1810772

[2] Higham CE (2016) Hypopituitarism Lancet 388:2403–2415 27041067 10.1016/S0140-6736(16)30053-8

[3] Nishioka H (2017) Recent evolution of endoscopic endonasal surgery for treatment of Pituitary Adenomas. Neurol Med Chir (Tokyo) 57:151–158 28239067 10.2176/nmc.ra.2016-0276 PMC5409268

[4] Lake MG (2013) Pituitary adenomas: an overview. Am Fam Physician 88:319–327 24010395

[5] Gondim JA (2009) Headache associated with pituitary tumors. J Headache Pain 10:15–20 19067118 10.1007/s10194-008-0084-0 PMC3451766

[6] Tahara S (2022) An Overview of Pituitary Incidentalomas: Diagnosis, Clinical Features, and Management. Cancers (Basel). 14 10.3390/cancers14174324 PMC9454484 36077858

[7] Wolf A (2016) Quantitative evaluation of headache severity before and after endoscopic t ranssphenoidal surgery for pituitary adenoma. J Neurosurg 124:1627–1633 26495954 10.3171/2015.5.JNS1576

[8] Jang MK (2020) Prevalence and Impact of Postoperative Headaches in nonfunctioning Pituitary Macroadenoma patients: a longitudinal cohort study. World Neurosurg 133:e633–e639 31604133 10.1016/j.wneu.2019.09.123

[9] Ahmed F (2012) Headache disorders: differentiating and managing the common subtypes. Br J Pain 6:124–132 26516483 10.1177/2049463712459691 PMC4590146

[10] Hayashi Y (2016) Significant improvement of intractable headache after transsphenoidal surgery in patients with pituitary adenomas; preoperative neuroradiological evaluation and intraoperative intrasellar pressure measurement. Pituitary 19:175–182 26659379 10.1007/s11102-015-0696-8

[11] Fleseriu M (2009) Significant headache improvement after transsphenoidal surgery in patients with small sellar lesions. J Neurosurg 110:354–358 19012490 10.3171/2008.8.JNS08805

[12] Siegel S (2017) Presence of headache and headache types in patients with tumors of the sellar region-can surgery solve the problem? Results of a prospective single center study. Endocrine 56:325–335 28243973 10.1007/s12020-017-1266-9

[13] Schankin CJ (2012) Headache in patients with pituitary adenoma: clinical and paraclinical findings. Cephalalgia 32:1198–1207 23059488 10.1177/0333102412462639

[14] Jakobsson S (2020) Extended support within a person-centered practice after surgery for patients with pituitary tumors: protocol for a quasiexperimental study. JMIR Res Protoc 9, e17697 10.2196/17697 PMC7404015 32706741

[15] Andersson A (2022) Headache before and after endoscopic transsphenoidal pituitary tumor surgery: a prospective study. J Neurol Surg B Skull Base 83:e360–e366 35832989 10.1055/s-0041-1729180 PMC9272269

[16] Delport R (2023) Headache improvement following endoscopic resection of Pituitary Adenomas. World Neurosurg 176:e456–e461 37277024 10.1016/j.wneu.2023.05.082

[17] Stewart WF (2001) Development and testing of the Migraine Disability Assessment (MIDAS) Questionnaire to assess headache-related disability. Neurology 56:S20–28 11294956 10.1212/wnl.56.suppl_1.s20

[18] Stewart WF (2000) Validity of the Migraine Disability Assessment (MIDAS) score in comparison to a diary-based measure in a population sample of migraine sufferers. Pain 88:41–52 11098098 10.1016/S0304-3959(00)00305-5

[19] Lipton RB (2001) Clinical utility of an instrument assessing migraine disability: the Migraine Disability Assessment (MIDAS) questionnaire. Headache 41:854–861 11703471

[20] Edmeads J (2001) Potential of the Migraine Disability Assessment (MIDAS) Questionnaire as a public health initiative and in clinical practice. Neurology 56:S29–34 11294957 10.1212/wnl.56.suppl_1.s29

[21] Torres-Ferrus M (2020) Influence of headache pain intensity and frequency on migraine-related disability in chronic migraine patients treated with OnabotulinumtoxinA. J Headache Pain 21:88 32652924 10.1186/s10194-020-01157-8 PMC7353810

[22] Asa SL, Ezzat S (2009) The pathogenesis of pituitary tumors. Annu Rev Pathol 4:97–126 19400692 10.1146/annurev.pathol.4.110807.092259

[23] EuroQol–a new facility for the measurement of health-related quality of life. Health Policy 16, 199–208 (1990) 10.1016/0168-8510(90)90421-9 10109801

[24] Rabin R, de Charro F (2001) EQ-5D: a measure of health status from the EuroQol Group. Ann Med 33:337–343 11491192 10.3109/07853890109002087

[25] Sommerfelt H (2019) Impact of transsphenoidal surgery for pituitary adenomas on overall health-related quality of life: a longitudinal cohort study. Br J Neurosurg 33:635–640 31544528 10.1080/02688697.2019.1667480

[26] Hallén T (2022) Sinonasal symptoms and self-reported health before and after endoscopic pituitary Surgery-A prospective study. J Neurol Surg B Skull Base 83:e160–e168 35832966 10.1055/s-0041-1722929 PMC9272326

[27] Stafford MR (2012) EQ-5D™-derived utility values for different levels of migraine severity from a UK sample of migraineurs. Health Qual Life Outcomes 10:65 22691697 10.1186/1477-7525-10-65 PMC3407525

[28] Xu R (2011) EuroQol (EQ-5D) health utility scores for patients with migraine. Qual Life Res 20:601–608 21063786 10.1007/s11136-010-9783-5

[29] WHO.: WHO Classification of Tumours Editorial Board: Endocrine and Neuroendocrine tumours, vol. 8. 5th edn (2022) (2022)

[30] Rizzoli P (2016) Headache in patients with Pituitary lesions: a longitudinal cohort study. Neurosurgery 78:316–323 26485333 10.1227/NEU.0000000000001067

[31] Suri H, Dougherty C (2018) Clinical presentation and management of Headache in Pituitary tumors. Curr Pain Headache Rep 22:55 29904889 10.1007/s11916-018-0710-8

[32] Taylor LP (2014) Mechanism of brain tumor headache. Headache 54:772–775 24628259 10.1111/head.12317

[33] Levy MJ (2004) Pituitary volume and headache: size is not everything. Arch Neurol 61:721–725 15148150 10.1001/archneur.61.5.721

[34] Yu B (2017) Clinical characteristics and risk factors for headache associated with non-functioning pituitary adenomas. Cephalalgia 37:348–355 27154998 10.1177/0333102416648347

[35] Simander G (2022) Intrasellar pressure in patients with pituitary adenoma - relation to tumour size and growth pattern. BMC Neurol 22:82 35264140 10.1186/s12883-022-02601-9 PMC8905730

[36] Levy MJ (2005) The clinical characteristics of headache in patients with pituitary tumours. Brain 128:1921–1930 15888539 10.1093/brain/awh525

[37] Sauro KM (2010) HIT-6 and MIDAS as measures of headache disability in a headache referral population. Headache 50:383–395 19817883 10.1111/j.1526-4610.2009.01544.x

